# Diagnostic value of HHV-6A/B genotyping in immunocompromised adults

**DOI:** 10.1128/jcm.01898-25

**Published:** 2026-06-29

**Authors:** Virginia Rodríguez-Lorente, Paola Charry, Ares Guardia, Francesc Fernández-Avilés, Montserrat Rovira, María Queralt Salas, Carmen Martínez, Laura Rosiñol, Marta Bodro, Ana Belén Pérez, Isabel Machuca, Ana Cervilla, María del Mar Mosquera, Miguel J. Martínez, María Suárez-Lledó, María Ángeles Marcos

**Affiliations:** 1 Microbiology Department, Hospital Clínic de Barcelona, Universitat de Barcelona, Fundació de Recerca Clínic Barcelona-Institut d'Investigacions Biomèdiques August Pi Sunyer (FRCB-IDIBAPS) 146245, Barcelona, Spain; 2 Hematology Department, Hospital Clínic de Barcelona, IDIBAPS 146245 , Barcelona, Spain; 3 Infectious Diseases Department, Hospital Clínic de Barcelona, Universitat de Barcelona, IDIBAPS 16719, Barcelona, Spain; 4 Centro de Investigación Biomédica en Red de Enfermedades Infecciosas (CIBERINFEC), CIBER-ISCIII, Instituto de Salud Carlos III 38176, Madrid, Spain; 5 Microbiology Department, Hospital Universitario Reina Sofía, Instituto Maimónides de Investigación Biomédica de Córdoba (IMIBIC) 16501, Córdoba, Spain; 6 Infectious Diseases Department, Hospital Universitario Reina Sofía, Universidad de Barcelona, IMIBIC 16501, Córdoba, Spain; 7 Microbiology Department, Hospital Clinic of Barcelona 740670, Barcelona, Spain; 8 Microbiology Department, Hospital Clínic de Barcelona, Universitat de Barcelona, Instituto de Salud Global de Barcelona (ISGlobal) 16719, Barcelona, Spain; St Jude Children's Research Hospital, Memphis, Tennessee, USA

**Keywords:** human herpesvirus 6A, human herpesvirus 6B, endogenous human herpesvirus 6, DNA genotyping, real-time PCR

## Abstract

Human herpesvirus 6 (HHV-6) comprises two genetically and biologically distinct species, HHV-6A and HHV-6B, yet species-level differentiation is rarely performed in clinical practice. We conducted a retrospective study of 119 immunocompromised adults with detectable HHV-6 DNA between 2020 and 2025 to characterize species distribution, compare epidemiological, clinical, and virological features, and evaluate the diagnostic relevance of genotyping. Viral species were detected by real-time polymerase chain reaction (PCR) across multiple sample types, and suspected chromosomal integration was confirmed through detection of endogenous HHV-6 (eHHV-6) DNA in hair follicles by quantitative real-time PCR. HHV-6B accounted for ~96% of infections and was significantly associated with organ involvement, predominantly gastrointestinal disease, irrespective of plasma viral load. In contrast, HHV-6A was less frequently detected in tissues and was consistently associated with viremia and chromosomal integration, independent of viral load. Genotypic concordance across plasma, biopsy, cerebrospinal fluid, bronchoalveolar lavage, and hair follicle samples from the same patient was complete. Incorporating species-level genotyping and targeted testing for chromosomal integration (eHHV-6) into diagnostic workflows may improve interpretation of viral detection and help prevent unnecessary antiviral therapy.

**IMPORTANCE:**

Species-specific identification of human herpesvirus 6 (HHV-6A/B) has critical diagnostic implications in immunocompromised patients, although it is rarely performed in diagnostic laboratories. Our study showed that HHV-6A detection is associated with chromosomal integration, whereas HHV-6B is the main species linked to clinically relevant disease and invasive tissue infection. This study emphasizes that implementing species-specific PCR and testing for eHHV-6 in clinical microbiology laboratories may help interpret viral DNA detection, prevent misclassification of integrated virus as active infection, and avoid unnecessary antiviral therapy.

## INTRODUCTION

Human herpesvirus 6 (HHV-6) belongs to the *Herpesviridae* family, *Betaherpesvirinae* subfamily, and *Roseolovirus* genus. Despite their high overall genetic sequence identity (~90%), in 2012 they were classified as two distinct virus species, HHV-6A and HHV-6B, by the International Committee on Taxonomy of Viruses (1), owing to differences in biological, epidemiological, immunological, and genetic properties, as well as clinical implications (2, 3). Differentiating between HHV-6A and HHV-6B is therefore useful for diagnosis, treatment strategies, and understanding of disease outcomes.

During viral entry into human cells, HHV-6A uses the CD46 receptor (membrane cofactor protein), while HHV-6B uses CD134 (also known as OX40), although occasional CD46 usage has been described (4–6). CD46 is expressed on all nucleated cells (e.g., T and B lymphocytes, monocytes/macrophages, epithelial and endothelial cells, glial cells, neurons) (7), whereas CD134 is predominantly expressed on activated CD4^+^ T lymphocytes (5). Following primary infection, both species can latently persist in various anatomical sites and cell types, particularly monocytes-macrophages (8).

HHV-6 infection usually occurs during infancy and early childhood. In immunocompetent individuals, both species are typically mild and self-limiting; however, in immunosuppressed patients, HHV-6A/B can act as an opportunistic pathogen, and its reactivation has been associated with serious diseases, with HHV-6B being the most commonly associated (9).

HHV-6 is the only herpesvirus known to integrate into human chromosomes *in vivo*. Previously referred to as chromosomally integrated HHV-6 (ciHHV-6), it is now commonly termed endogenous HHV-6 (eHHV-6) (10). Integration occurs in the subtelomeric region of chromosomes (3, 11) and has been described in approximately 1% of the general population (12–16). The virus can be genetically transmitted, and offspring harbor one copy of the viral genome in every nucleated cell. Confirmation of eHHV-6 can be done by testing for HHV-6 DNA using real-time polymerase chain reaction (PCR) in a hair follicle or nail (17, 18), as only eHHV-6 carriers have HHV-6 DNA in these cells, or by digital PCR on whole blood and samples that contain cells with integrated DNA (15).

Currently, PCR is the technique of choice for detecting HHV-6A/B in plasma and even in highly cellular samples (e.g., tissue biopsies), as immunohistochemistry and *in situ* hybridization remain limited for confirming active infection. In recent years, a marked increase in HHV-6A/B replication has been observed in our setting, with a large proportion of affected patients requiring antiviral therapy (19–21). This increase appears to be related to the growing population of immunosuppressed individuals and broader use of intensive immunosuppressive regimens. Among these patients, allogeneic hematopoietic stem cell transplantation (allo-HSCT) recipients present the highest incidence of HHV-6A/B reactivation, a trend associated with the widespread use of post-transplant cyclophosphamide (PTCy) for prophylaxis of graft-versus-host disease (GVHD) and the incorporation of letermovir (LET) for cytomegalovirus (CMV) prophylaxis. While LET has markedly reduced the incidence of CMV infection, it has not been shown to be effective in preventing HHV-6 infection (22). Consequently, HHV-6 has emerged as an increasingly relevant pathogen in the current post-transplant setting, often requiring antiviral intervention.

However, despite its potential clinical relevance, routine distinction between HHV-6A and HHV-6B remains uncommon in diagnostic laboratories, and the clinical implications of species-level identification in immunocompromised adults are not well established. The main objective of this study was to identify the viral species HHV-6A and HHV-6B in immunosuppressed adults with viral replication. Secondary objectives included the description and comparison of epidemiological, clinical, and virological characteristics and evaluation of the diagnostic relevance of species-level genotyping.

## MATERIALS AND METHODS

### Study design and patients

We conducted a retrospective study of HHV-6 infection in immunocompromised adults, in whom HHV-6 testing was requested according to clinical criteria rather than a predefined protocol. A total of 119 patients aged ≥18 years with a history of immunosuppression and HHV-6 detection in any clinical specimen between November 2020 and May 2025 were included from two participating centers. The history of immunosuppression included allo-HSCT, autologous HSCT (auto-HSCT), or ongoing therapy with immunosuppressive agents for an underlying disease.

All HHV-6-positive samples used for viral detection and quantification were stored at –80°C in the Microbiology Department of the Hospital Clínic. One representative sample of each available type per patient was selected for the genotyping study. Sample types included plasma, peripheral blood mononuclear cells (PBMCs), tissue biopsy, hair follicle, cerebrospinal fluid (CSF), and bronchoalveolar lavage (BAL). HHV-6 PCR testing was performed in plasma samples from patients presenting fever, cytopenia, and/or skin rash; in plasma and CSF samples from patients with neurological symptoms; and in plasma and BAL samples from patients with respiratory symptoms. HHV-6 PCR was performed in biopsy specimens, in the presence of symptoms in other organs (hepatitis or gastrointestinal).

Testing for eHHV-6 was performed in patients with high HHV-6 viral loads detected across multiple tissues in addition to plasma or when the viral loads remained stable despite appropriate antiviral therapy.

Organ involvement was defined by compatible clinical manifestations together with a positive HHV-6 PCR result in the corresponding specimen, negative results for Epstein-Barr virus and CMV, and exclusion of GVHD.

Antiviral treatment was initiated in patients with evidence of HHV-6 replication and compatible clinical manifestations. When HHV-6 replication was only detected in plasma, therapy was considered only in cases showing a progressive increase in viral load. The choice of antiviral agent was guided by clinical judgment.

### HHV-6 detection and quantification

HHV-6 detection and quantification were performed by quantitative real-time PCR using the ELITe InGenius system (ELITechGroup, Italy) and were optimized for the isolation of DNA from 200 µL samples. ELITe InGenius contains a combination of lytic and extraction reagents designed to perform cell lysis and DNA extraction. The DNA was eluted using 100 μL of elution buffer. The real-time PCR amplification was performed according to the manufacturer’s instructions.

Viral load was expressed in international units per milliliter (IU/mL) for plasma and in copies per milliliter (copies/mL) for all other sample types. For plasma, the conversion factor between copies/mL and IU/mL was 1.10. In tissue biopsies, viral load was normalized as copies per 10⁵ cells.

Biopsy and hair follicle samples were pre-digested overnight with proteinase K and TRIS-EDTA buffer at 56°C and 550 rpm in an Eppendorf ThermoMixer thermoblock (Eppendorf, Germany). PBMCs were isolated from whole blood by centrifugation using a Lymphoprep density gradient medium (STEMCELL Technologies, Canada). DNA concentration in biopsies was measured using the NanoDrop 2000-2000C UV-Vis spectrophotometer (Thermo Fisher Scientific, USA). The value was expressed in nanograms per microliter. Subsequently, the viral load was calculated in copies per 10^5^ cells, following the manufacturer’s instructions (ELITe InGenius). All remaining samples and extracts were stored at −80°C for subsequent analysis.

### Genotyping study

HHV-6A/B genotyping was performed using DNA extracts previously obtained with ELITe InGenius system. Species differentiation was achieved by qualitative real-time PCR with the Altostar HHV6 PCR Kit 1.5 (altona Diagnostics, Germany) on a LightCycler 480 SW 2.0 instrument (Roche Diagnostics, Switzerland), according to the manufacturer’s instructions.

### Data collection and statistics

Clinical and laboratory data collected from each patient were stored in the database developed for this study. IBM SPSS version 26.0 (IBM Corp., Armonk, NY, USA) was used for the statistical analyses. The cohort was categorized into two groups: patients with HHV-6A and patients with HHV-6B. We reported the number and percentage of patients for categorical variables and the median (first quartile; third quartile) for continuous variables. The assumption of normality was checked using the Shapiro-Wilk test. Categorical variables were compared using the chi-squared test or Fisher’s exact test, as appropriate. The Mann-Whitney *U*-test was used for comparisons of continuous variables between two independent groups. For graphical representation of viral load distributions, individual values were plotted in scatter plots, with a log10 scale on the *y*-axis to facilitate visualization of the wide range of values. The level of significance was set at 0.05 (two-tailed).

## RESULTS

### Study population and samples

Baseline demographic, clinical, and virological characteristics of the 119 immunosuppressed patients with HHV-6 detected are shown (Table 1). The median age was 52 years (interquartile range, 42–64), and 50.4% of patients were male. Eighty-two patients (68.9%) underwent HSCT, including 73 (61.3%) who received allo-HSCT and 9 auto-HSCT (7.6%). Among underlying conditions, 37 patients (31.1%) received other immunosuppressive treatments, such as cytotoxic chemotherapy (*n* = 18), immunotherapy (*n* = 6), chimeric antigen receptor T-cell (CAR-T) therapy (*n* = 4), gene therapies (*n* = 1), or other treatments such as corticosteroids, cyclosporine, mycophenolate, and azathioprine (*n* = 8).

**TABLE 1:** Baseline, clinical, and virological characteristics of the cohort, categorized by HHV-6A and HHV-6B genotypes^*f*^

Patient characteristics	Total (*n* = 119)	HHV-6A (*n* = 5)	HHV-6B (*n* = 114)	*P*-value^*a*^
Age, yr (median, IQR)	52.0 (42.0–64.0)	42.0 (35.0–46.0)	53.0 (43.0–64.0)	0.057
Gender, *n* (%)				
Male	60 (50.4)	4 (80.0)	56 (49.1)	0.36
Female	59 (49.6)	1 (20.0)	58 (50.9)	
Type of immunosuppression, *n* (**%**)				
Allogeneic HSCT	73 (61.3)	2 (40.0)	71 (62.3)	0.452
Autologous HSCT	9 (7.6)	1 (20.0)	8 (7.0)	
Immunosuppressive treatment	37 (31.1)	2 (40.0)	35 (30.7)	
Location of HHV-6 detection, *n* (%)				
Organic	65 (54.6)	0 (0.0)	65 (57.0)	0.007
Viremia	19 (16.0)	3 (60.0)	16 (14.0)	
Viremia and organic	35 (29.4)	2 (40.0)	33 (29.0)	
Organ detection, *n* (%)**^*b*^**				
Gastrointestinal	91 (91.0)	1 (50.0)	90 (92.0)	0.17
Gastrointestinal and cardiac	1 (1.0)	0 (0.0)	1 (1.0)	
Gastrointestinal and respiratory	2 (2.0)	0 (0.0)	2 (2.0)	
Gastrointestinal and CNS	1 (1.0)	0 (0.0)	1 (1.0)	
Respiratory	1 (1.0)	0 (0.0)	1 (1.0)	
CNS	4 (4.0)	1 (50.0)	3 (3.0)	
HHV-6 detection in hair follicle, *n* (%)				
No	109 (91.6)	0 (0.0)	109 (95.6)	< 0.001
Yes	10 (8.4)	5 (100.0)	5 (4.4)	
Clinical symptoms, *n* (%)				
Asymptomatic	28 (23.5)	3 (60.0)	25 (21.9)	0.098
Encephalitis	4 (3.4)	1 (20.0)	3 (2.6)	
Fever	13 (11.0)	0 (0.0)	13 (11.4)	
Fever and rash	1 (0.8)	0 (0.0)	1 (0.9)	
Fever, rash, and cytopenia	1 (0.8)	0 (0.0)	1 (0.9)	
Gastrointestinal	71 (59.7)	1 (20)	70 (61.4)	
Gastrointestinal and respiratory	1 (0.8)	0 (0.0)	1 (0.9)	
Antiviral treatment, *n* (%)				
No	61 (51.3)	2 (40.0)	59 (51.8)	0.67
Yes	58 (48.7)	3 (60.0)	55 (48.2)	
Type of antiviral treatment, *n* (%)**^*c*^**				
GCV/VGCV	28 (48.3)	2 (66.7)	26 (47.3)	0.43
FOS	13 (22.4)	0 (0.0)	13 (23.6)	
CDV	1 (1.7)	0 (0.0)	1 (1.8)	
More than one antiviral	16 (27.6)	1 (33.3)^*d*^	15 (27.3)^*e*^	
CMV prophylaxis with LET, *n* (**%**)				
No	72 (60.5)	4 (80.0)	68 (59.6)	0.65
Yes	47 (39.5)	1 (20.0)	46 (40.4)	
CMV infection, *n* (**%**)				
No	101 (84.9)	5 (100.0)	96 (84.2)	1.00
Yes	18 (15.1)	0 (0.0)	18 (15.8)	

^*a*^ Due to the small sample size and low expected frequencies in several categories, preferentially used for categorical comparisons, and Mann-Whitney *U*-test were applied for continuous variables.

^*b*^ Percentage calculated from the total number of patients with organ detection [*n* (total) = 100; *n* (HHV-6A) = 2; *n* (HHV-6B) = 98].

^*c*^ Percentage calculated from the total number of patients who received antiviral treatment [*n* (total) = 58; *n* (HHV-6A) = 3; *n* (HHV-6B) = 55].

^*d*^ GCV/VGCV, FOS, CDV.

^*e*^ FOS, CDV (*n* = 4), GCV/VGCV, FOS (*n* = 6), GCV/VGCV, CDV (*n* = 2), GCV/VGCV, FOS, CDV (*n* = 3).

^*f*^ GCV/VGCV, ganciclovir or valganciclovir; CNS, central nervous system; FOS, foscarnet; CDV, cidofovir; CMV, cytomegalovirus; LET: letermovir; HSCT, hematopoietic stem cell transplantation; IQR, interquartile range.

Twenty-eight (23.5%) patients were asymptomatic, whereas 72 patients (60.5%) presented gastrointestinal symptoms. Antiviral therapy for HHV-6 infection was administered to 58 patients (48.7%), of whom 16 (13.5%) received more than one antiviral agent. Ganciclovir or valganciclovir was the most frequently administered (48.3%). CMV co-infection occurred in 18 (15.8%) patients, 12 (66.6%) of whom had undergone allo-HSCT. LET prophylaxis was administered to 47 allo-HSCT patients (39.5%), with CMV infection documented in only one patient.

HHV-6 was detected in organ samples in 100 patients (84%), and viremia was observed in 54 patients (45.4%). Viral detection in hair follicles, consistent with eHHV-6, was identified in 10 patients (8.4%).

### HHV-6 genotyping

HHV-6B was detected in 114 patients (95.8%) and HHV-6A in 5 (4.2%) (Table 1). HHV-6A was identified only in patients with viremia (60.0%) or with both viremia and organ involvement (40.0%), while none of the HHV-6A cases showed only organ detection. In contrast, HHV-6B infection was predominantly associated with organ involvement (86.0%), with gastrointestinal involvement being the most frequent organ manifestation (96.0%). This distribution was statistically significant (*P* = 0.007). Chromosomal integration confirmed in hair follicles was significantly more frequent in HHV-6A (100% vs 4.4%; *P* < 0.001).

HHV-6A/B genotyping was performed in multiple sample types from the same patient. In total, 168 HHV-6-positive samples (95 biopsies, 57 plasma, 10 hair follicles, 4 CSF, 1 PBMC, 1 BAL) from 119 immunosuppressed patients were included for HHV-6A/B genotyping (Table S1). All the samples from a given patient showed the same viral genotype. HHV-6A DNA was detected in all tissue samples studied from patients in whom HHV-6A was also detected in hair follicles.

**Fig 1:**
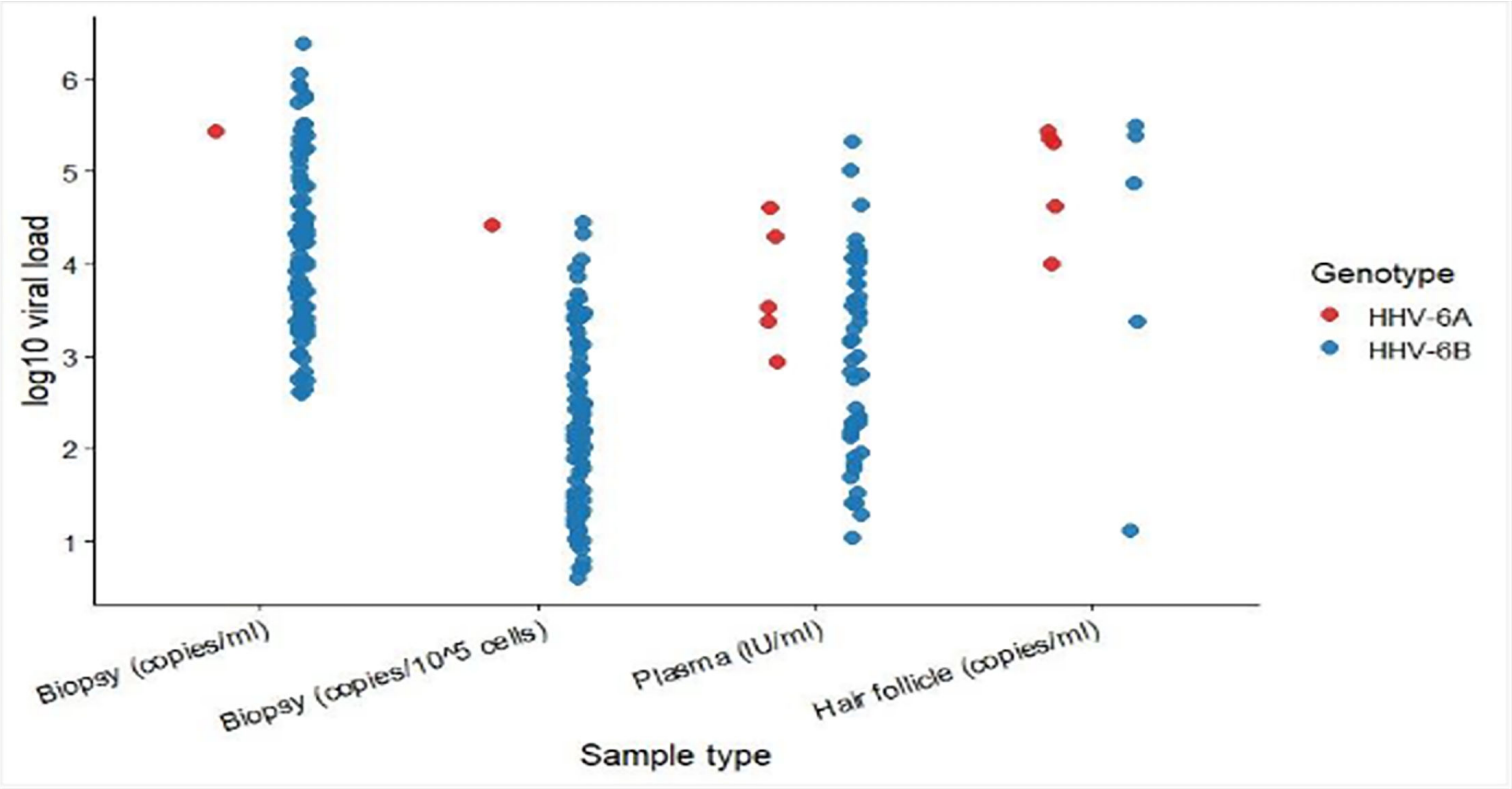
HHV-6 viral load values in biopsy, plasma, and hair follicle samples, stratified by viral species (HHV-6A and HHV-6B).

Plasma viral loads were comparable between HHV-6A and HHV-6B, although HHV-6A tended to show higher values across sample types without reaching statistical significance. Figure 1 shows the distribution of viral loads across sample types and HHV-6 genotypes.

## DISCUSSION

In this study, we genotyped HHV-6A and HHV-6B in 119 immunocompromised adult patients with HHV-6 detected and provided a comparative overview of the epidemiological, clinical, and virological features. HHV-6B was overwhelmingly predominant (~96%) and was mainly associated with organ involvement, while HHV-6A was uncommon and detected only in patients with viremia and chromosomal integration.

Although studies performing HHV-6A/B genotyping in immunocompromised adults are limited, the clear predominance of HHV-6B in this cohort aligns with published data reporting HHV-6B in 85%–95% of active infections among allo-HSCT recipients and other severely immunosuppressed adults (2, 22).

HHV-6B reactivation is closely related to T-cell dysfunction, particularly in transplant settings where PTCy and prolonged lymphopenia are common (22, 23). In our cohort, HHV-6B was strongly associated with organ involvement—predominantly gastrointestinal—consistent with its known tropism for activated CD4^+^ T cells through CD134 (5). This receptor-restricted entry contributes to replication focused on lymphoid, epithelial, and glandular tissues, which explains the characteristic clinical picture of fever, gastrointestinal involvement, cytopenia, and occasionally encephalitis described in previous studies (8, 16, 24, 25). Importantly, HHV-6B infection occurred at moderate plasma viral loads, supporting the view that DNAemia alone does not predict disease severity and should be interpreted in conjunction with clinical and organ-specific findings, as recommended by the European Conference on Infections in Leukaemia (ECIL-7) guidelines (16).

HHV-6A showed a different pattern. It was always detected in plasma and was never detected as an isolated organ infection. All five HHV-6A cases occurred in individuals with eHHV-6, confirmed by PCR in hair follicles. These findings are consistent with reports indicating that HHV-6A in adults is most often identified as eHHV-6, rather than as a reactivating virus (3, 26). We observed a prevalence of eHHV-6 of 8.4%, which is higher than previously described (26). HHV-6A also tended to show higher viral loads than HHV-6B across various sample types (plasma, biopsy, CSF, PBMCs), although the difference was not statistically significant. The lack of statistically significant differences in hair follicle viral load between HHV-6A and HHV-6B should be interpreted with caution, as this comparison was restricted to a very small subset of cases (*n* = 5 per group), which substantially limits statistical power. Therefore, the absence of a statistically significant difference in hair follicle viral load does not contradict the observed association between HHV-6A detection and chromosomal integration. Persistently high HHV-6 DNA levels in several compartments, including plasma, CSF, and biopsies, without clinical disease, are characteristic of chromosomal integration (3, 8, 15, 26, 27). These observations highlight the diagnostic difficulties associated with eHHV-6A in immunosuppressed patients, in whom high-level DNAemia may be misinterpreted as viral reactivation. Failure of plasma viral load to decrease despite therapy, or persistent detection in multiple tissues in the absence of symptoms, should raise the suspicion of eHHV-6 and prompt confirmatory testing in hair follicles or nails (3, 26).

An important aspect of this study is the systematic evaluation of multiple sample types per patient—including plasma, biopsies, CSF, BAL, and hair follicles. We found perfect intra-patient concordance of HHV-6 genotype regardless of the sample type. This provides clinically actionable evidence that, once a genotype is established in any compartment, replication detected elsewhere can be interpreted within the same species-specific framework.

Among the main strengths of this study is the differentiation between HHV-6A and HHV-6B in a large cohort of immunocompromised adult patients, an aspect that is rarely specifically addressed. Furthermore, the inclusion of multiple sample types (biopsy, CSF, BAL, PBMCs, and hair follicles), distinct from the most frequently studied sample (plasma), allowed confirming intra-patient genotypic concordance and distinguishing between active infection and integrated virus. Conversely, given the retrospective nature of this study, the clinical course, response to antiviral treatment, and causality of mortality attributable to HHV-6 infection could not be assessed in our cohort. In addition, most patients were recruited from a single center, which may limit the generalizability of the findings. However, our results are consistent with respect to laboratory and diagnostic methods, as well as the clinical criteria applied, thereby supporting the robustness of our interpretations. Another limitation is the small number of HHV-6A cases, although this reflects the proportion observed in our cohort. This limitation may restrict comparative analysis and reduce the ability to detect specific clinical associations. Furthermore, the lack of longitudinal clinical data precludes assessment of a potential pathogenic role of HHV-6A in this cohort.

In conclusion, in this genotype-stratified cohort, HHV-6B was the predominant and clinically relevant species in immunocompromised adults, mainly associated with organ disease. In contrast, detection of HHV-6A largely reflected chromosomal integration rather than active infection. Incorporation of species-level genotyping and targeted testing for eHHV-6 into diagnostic algorithms for immunocompromised patients, particularly HSCT recipients, may improve clinical interpretation and help avoid unnecessary antiviral therapy. Further studies including a larger number of centers are warranted to confirm these findings and to evaluate their relevance in other clinical settings, including solid organ transplantation and CAR-T therapy.

## SUPPLEMENTAL MATERIAL

Table s1 jcm.01898-25-s0001.docx Total number of samples analyzed in 5 patients with HHV-6A and 114 patients with HHV-6B.

ASM does not own the copyrights to Supplemental Material that may be linked to, or accessed through, an article. The authors have granted ASM a non-exclusive, world-wide license to publish the Supplemental Material files. Please contact the corresponding author directly for reuse.
