# Supplementary material for: Diagnostic value of HHV-6A/B genotyping in immunocompromised adults
Source: J Clin Microbiol. 2026 Jun 29;64(8):e01898-25. doi: 10.1128/jcm.01898-25 (PMC13463904; doi:10.1128/jcm.01898-25)
Supplement: Supplementary file 1 [file jcm.01898-25-s0001.docx]

**SUPPLEMENTAL MATERIAL**

| **Table S1.** Viral load of positive samples analyzed in the cohort, categorized by the HHV-6A and HHV-6B genotypes. | | | | | |
| --- | --- | --- | --- | --- | --- |
| *Samples characteristics* | **Total (n = 168)** | **HHV-6A (n = 13)** | **HHV-6B (n = 155)** | **p value^a^** |  |
| **Viral load (median and IQR)** |  |  |  |  |  |
| Biopsy (copies/ml) | 1.69×10⁴ (2.58×10³ - 7.18×10⁴) | 2.64×10⁵ | 1.64×10⁴ (2.58×10³–6.85×10⁴) | 0.179 |  |
| Biopsy (copies/10^5^ cells) | 1.40×10² (2.50×10¹ - 7.30×10²) | 2.56×10⁴ | 1.36×10² (2.40×10¹–6.62×10²) | 0.267 |  |
| Plasma (IU/ml)^b^ | 1.46×10³ (1.83×10² - 6.28×10³) | 1.33×10³ (2.34×10² - 1.94×10⁴) | 1.22×10³ (1.57×10¹–6.17×10³) | 0.431 |  |
| Hair follicle (copies/ml) | 7.42×10⁴ (9.90×10³ - 2.39×10⁵) | 1.35×10⁵ (2.61×10⁴ - 2.46×10⁵) | 7.42×10⁴ (2.40×10³–2.39×10⁵) | 0.121 |  |
| CSF (copies/ml) | 1.53×10⁴ (7.79×10³ - 3.54×10⁵) | 2.05×10⁵ | 1.53×10⁴ (7.79×10³–3.54×10⁵) |  |  |
| PBMCs (copies/ml) | 5.73×10⁶ | 5.73×10⁶ | - |  |  |
| BAL (copies/ml) | 1.46×10⁵ | - | 1.46×10⁵ |  |  |
| n, total number of samples analyzed in 5 patients with HHV-6A and 114 patients with HHV-6B  IQR: interquartile range; CSF: cerebrospinal fluid; PBMCs: peripheral blood mononuclear cells; BAL: bronchoalveolar lavage  a, p-value were calculated using Mann-Whitney U test  b, conversion factor of copies/ml to IU/ml was 1.10 | | | | | |
